# Measuring vital signs in children with fever at the emergency department: an observational study on adherence to the NICE recommendations in Europe

**DOI:** 10.1007/s00431-020-03601-y

**Published:** 2020-02-08

**Authors:** Josephine van de Maat, Hein Jonkman, Elles van de Voort, Santiago Mintegi, Alain Gervaix, Silvia Bressan, Henriette Moll, Rianne Oostenbrink

**Affiliations:** 1 Department of General Paediatrics, Erasmus MC – Sophia Children’s Hospital, P.O. Box 2060, 3000 CB Rotterdam, The Netherlands; 2 Paediatric Emergency Department, Cruces University Hospital, Plaza de Cruces s/n, 48903 Bilbao, Spain; 3 Department of Paediatrics, University Hospital of Geneva, Rue Willy Donze 6, 1205 Geneva, Switzerland; 4 University Hospital of Padua, Padua, Italy

**Keywords:** Professional practice, Guideline adherence, Emergency medical services, Triage, Child, Child, Preschool, Infections, Pediatrics

## Abstract

Vital signs can help clinicians identify children at risk of serious illness. The NICE guideline for fever in under-fives recommends a routine measurement of temperature, heart rate, capillary refill and respiratory rate in all febrile children visiting the emergency department (ED). This study aims to evaluate the measurement of paediatric vital signs in European EDs, with specific attention to adherence to this NICE guideline recommendation. In a prospective observational study, we included 4560 febrile children under 16 years from the ED of 28 hospitals in 11 European countries (2014–2016). Hospitals were academic (*n* = 17), teaching (*n* = 10) and non-teaching (*n* = 1) and ranged in annual paediatric ED visits from 2700 to 88,000. Fifty-four percent were male, their median age was 2.4 years (IQR 1.1–4.7). Temperature was measured most frequently (97%), followed by capillary refill (86%), heart rate (73%), saturation (56%) and respiratory rate (51%). In children under five (*n* = 3505), a complete measurement of the four NICE-recommended vital signs was performed in 48% of patients. Children under 1 year of age, those with an urgent triage level and with respiratory infections had a higher likelihood of undergoing complete measurements. After adjustment for these factors, variability between countries remained.

*Conclusion:* Measuring vital signs in children with fever in the ED occurs with a high degree of practice variation between different European hospitals, and adherence to the NICE recommendation is moderate. Our study is essential as a benchmark for current clinical practice, in order to tailor implementation strategies to different European settings.

**Table:** 

**What is Known:** *• Vital signs can quickly provide information on disease severity in children in the emergency department (ED), and the NICE guideline for fever in under-fives recommends to routinely measure temperature, heart rate, capillary refill and respiratory rate.* *• Data regarding measurement of vital signs in routine practice across European EDs is currently unavailable.*	
**What is New:** *• Measurement of vital signs in febrile children is highly variable across European EDs and across patient subgroups, and compliance to the NICE recommendation is <50%.* *• Children under 1 year of age, those with an urgent triage level and with respiratory infections had a higher likelihood of undergoing complete measurements.*

**Electronic supplementary material:**

The online version of this article (10.1007/s00431-020-03601-y) contains supplementary material, which is available to authorized users.

## Introduction

Fever is the most common reason for children to be brought to an emergency department (ED) [1–3], with causes ranging from self-limiting illnesses of childhood to serious bacterial infections (SBIs) that can prove fatal [3–5]. Vital signs can help clinicians identify children at risk of serious illness. Even though the level of evidence for the diagnostic accuracy of vital signs is varying, their importance is widely acknowledged [6]. Vital signs form the basis of paediatric early warning scores (PEWS) that are widely used to monitor disease severity of children in the inpatient setting [7]. Moreover, they are included in several prediction models for serious infections and in disease-specific guidelines for the ED setting [3, 8–12]. The NICE guideline for the assessment and initial management of fever in children under five recommends a routine measurement of temperature, heart rate, capillary refill and respiratory rate in all children presenting to the ED with a fever [13]. These recommendations have been adopted throughout a large number of European hospitals.

Not measuring vital signs may pose the patient at risk of underestimating the severity of illness and may delay appropriate treatment [14]. From adult research and single-country studies, we know that incomplete and inaccurate recording of vital signs is common [15–17]. This problem may be even larger in Europe, given the diversity of the countries, cultures and healthcare systems. However, international data on recording of vital signs across Europe in children are lacking [18]. Information on the measurement of vital signs is crucial in order to fuel research on serious illness and to target quality improvement initiatives in paediatric emergency medicine. This research aims to evaluate the current practice of measuring vital signs in febrile children in European EDs and, more specifically, the level of adherence to the NICE guideline recommendation to routinely measure four distinct vital signs.

## Methods

### Study design and population

We performed a prospective observational study in 28 EDs in 11 European countries, including patients under the age of 16 and with a fever as their presenting complaint. Children were excluded if they presented to the ED repeatedly for the same problem within 7 days, if they were treated with antibiotics in the 7 days before the ED visit, or if they had a documented allergy to antibiotics. For the current study, children with comorbidities were also excluded, as disease-specific characteristics may influence their management. In the whole population, we evaluated the measurement of vital signs. In children under five, we assessed the adherence to the recommendations to measure four distinct vital signs from the NICE guideline ‘Fever in under 5s: assessment and initial management’ [13].

### Data collection

Data collection took place between October 2014 and February 2016 within the network of Research in European Pediatric Emergency Medicine (REPEM). Detailed methods have been published earlier [19]. In short, all participating 28 EDs recorded medical information for all attending children with fever for one random day each month. We recorded general characteristics of patients (age, sex, weight, height, comorbidities), vital signs (heart rate, respiratory rate, temperature, oxygen saturation, capillary refill time) and information regarding diagnosis and management. Data were extracted from routine patient records, and filled in on an electronic study case report form (CRF) by the local investigator after the sampling day (Electronic Supplementary Material 1). Comorbidities and diagnoses were recorded according to pre-specified categories. We neither used ICD-codes for the recording of diagnoses nor had we access to data after the ED visit. Consequently, ‘diagnosis’ in this manuscript refers to a presumed diagnosis at ED discharge. All items in the CRF were mandatory to fill in, with the option to choose ‘unknown’. Unknown values on vital signs were seen as ‘not measured’, and were therefore considered to be outcomes rather than omissions. Local investigators were aware of the sampling days and the general scope of the study as a registry of febrile children, but vital sign measurement was not known as a specific point of interest. Hospital information was collected using a survey, including questions on guideline use. We collected data on hospital setting (inner city/rural/mixed), hospital type (academic/teaching/non-teaching), triage system and number of annual paediatric ED visits, similar to other studies on the organization of care (Electronic Supplementary Material 2) [20]. Setting reflects the population in the catchment area of the hospital. Academic hospitals are connected to a university, teaching hospitals non-university hospitals that provide training for paediatrics residents; non-teaching hospitals do not provide training of residents.

### Definitions

Not every study hospital used the same triage system, but they all classified children according to a five-point scale, ranging from ‘non-urgent’ to ‘immediate’, making comparisons possible. Owing to the small number of cases, patients in the ‘immediate’ and ‘very urgent’ categories were grouped together. Tachycardia and tachypnoea were defined according to the advanced pediatric life support (APLS) guideline [21]. Fever was defined as temperature ≥ 38 °C, hypoxia as peripheral oxygen saturation level of ≤ 94%. Crowding of the ED was defined for each hospital according to their number of total paediatric ED visits on the sampling day (less than usual/as usual/more than usual). We defined a usual number of total visits as the interquartile range of the number of total visits across the different sampling days per hospital. If on a sampling day the number of total visits was lower than the 25th percentile for that hospital, crowding was less than usual, if the number was higher than the 75th percentile, the ED was more crowded than usual.

Adherence to the NICE guideline was based on the following indicator: ‘*Measure and record temperature, heart rate, respiratory rate and capillary refill time as part of the routine assessment of a child with fever.*’ [13]*.* Adherence to the NICE guideline was defined as the complete measurements of those four vital signs in children under 5 years old.

### Statistical analysis

We used descriptive analyses to evaluate the frequency of measurement for all of the available vital signs in the study population. We examined practice variations between countries, age groups, triage levels and diagnoses, visualizing the measurement of vital signs by heat maps. We used diagnosis in these analyses as a proxy of presenting complaint (next to the fever) and suspicion of severity, assuming that children with RTIs would present with respiratory symptoms, enteric infections with vomiting or diarrhoea and that children with fever without source, urinary tract infections and sepsis/meningitis mostly present without specific symptoms but with a higher suspicion of invasive infections. We compared the frequency of detecting abnormal vital signs between countries that frequently measured vital signs and countries that measured them less often.

In assessing adherence to the NICE guideline, we measured the frequency of complete measurements in children under five from all hospitals that used the NICE recommendations. We tested the influence of age, triage level, diagnosis and crowding of the ED on adherence using a multilevel logistic regression model that included hospital as a random variable. Analyses were performed using SPSS (IBM, version 24) and R (version 3.5.2).

## Results

### Population characteristics

**Fig. 1:**
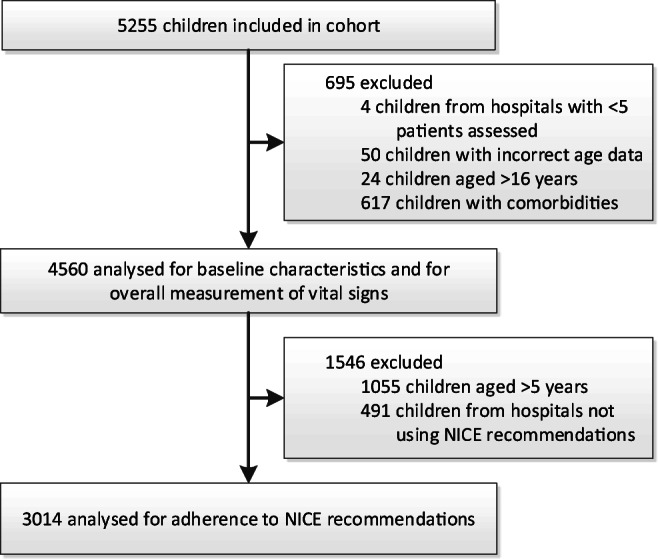
Flowchart of inclusion

In total, 5255 children were included in the complete cohort, all presenting with fever and without prior antibiotic treatment or repeated ED visits. In the current study, we included 4560 children. Exclusion was mostly because of comorbidities (Fig. 1). Of the included children, 53.8% were male and the median age was 2.4 years (interquartile range 1.1–4.7). Table 1 shows their baseline characteristics and provides information regarding patients’ way of referral and follow-up. Baseline characteristics of children with comorbidities have been published earlier [19]. In general, these children were more ill and older than children without comorbidities. Of the 28 participating hospitals, 17 were academic hospitals, 10 teaching hospitals and one non-teaching hospital (Table 2). They varied from inner city hospitals (*n* = 17) to regional (*n* = 2) and mixed hospitals (*n* = 9), and their number of annual paediatric ED visits ranged from 2700 to 88,000. Most hospitals used a local triage system (*n* = 8) or the Manchester triage system (*n* = 7, Table 2). All except the Spanish hospitals used the recommendation to routinely measure vital signs as mentioned in the NICE guideline.

**Table 1:** Baseline characteristics of the population, *n* = 4560

	*n*/*N* (%)^a^
General characteristics	
Male sex	2451/4557 (53.8%)
Age in years^b^	2.4 (1.1–4.7)
Season	
- Spring	1110/4560 (24.3%)
- Summer	766/4560 (16.8%)
- Autumn	1024/4560 (22.5%)
- Winter	1660/4560 (36.4%)
Way of referral	
- General practitioner	395/4524 (8.7%)
- Self	3966/4524 (87.7%)
- Other healthcare professional	163/4524 (3.6%)
Triage level	
- Immediate or very urgent	197/3850 (5.1%)
- Urgent	1042/3850 (27.1%)
- Standard	1866/3850 (48.5%)
- Non-urgent	745/3850 (19.4%)
Abnormal vital signs	
Fever (temperature ≥ 38 °C)	2403/4435 (54.2%)
Tachycardia^c^	1138/3341 (34.1%)
Tachypnoea^c^	665/2333 (28.5%)
Hypoxia (oxygen saturation ≤ 94%)	85/2567 (3.3%)
Prolonged capillary refill (> 3 s)	67/4560 (1.5%)
Disposition	
- Discharged home	4035/4559 (88.5%)
- Observation unit < 24 h	187/4559 (4.1%)
- Admitted to ward	321/4559 (7.0%)
- Admitted to ICU	11/4559 (0.2%)

^a^Unless stated otherwise

^b^Median (interquartile range)

^c^According to APLS guidelines

**Table 2:** Hospital information

Hospital	Country (code)	n	Annual PED visits	Type	Setting	Responsible specialist	Triage system	NICE recommendations on measurement of vital signs in use?
Aarhus Universitetshospital, Skejby	Denmark (DK)	24	5000	Academic	Mixed	Paediatrician	Local/national	Yes^a^
Hopital Antoine Béclère, Paris	France (FR)	53	25,000	Academic	Inner city	U	U	Yes^a^
Hôpital Mère-Enfant, Nantes		118	> 25,000	Academic	Inner city	Paediatrician	Local/national	
Hôpital Necker-Enfants malades, Paris		285	66,000	Academic	Inner city	PEM specialist	Local/national	
Hopital Robert Debre, Paris		384	88,000	Academic	Inner city	Paediatrician	U	
Roger Salengro Hospital, Lille		86	25,000	Teaching	Inner city	PEM specialist	MTS	
Heim Pal Children’s Hospital, Budapest	Hungary (HU)	111	30,000	Teaching	Mixed	Paediatrician	CTAS	Yes^a^
Meyer University Children’s Hospital, Florence	Italy (IT)	160	42,000	Academic	Inner city	Paediatrician	Local/national	Yes^b^
Ospedale dei Bambini, Azienda Ospedaliera Spedali Civili, Brescia		182	36,500	Academic	Mixed	Paediatrician	Local/national	
University Hospital, Padova		104	25,000	Academic	Inner city	Paediatrician	Local/national	
ErasmusMC—Sophia, Rotterdam	The Netherlands (NL)	60	4000	Academic	Inner city	Paediatrician	MTS	Yes^b^
Flevoziekenhuis, Almere		19	5000	Teaching	Mixed	Paediatrician	MTS	
Maasstad Ziekenhuis, Rotterdam		28	3500	Teaching	Inner city	Paediatrician	MTS	
Reinier de Graaf, Delft		29	2643	Teaching	Mixed	Paediatrician	MTS	
Sint Franciscus Ziekenhuis, Rotterdam		25	2700	Teaching	Inner city	Paediatrician	MTS	
Centro Hospitalar de Leiria, Leiria	Portugal (PT)	201	46,000	Teaching	Mixed	Paediatrician	Local/national	Yes^b^
Lisbon Medical Academic Center (Hospital de Santa Maria), Lisboa		282	50,000	Academic	Inner city	Paediatrician	Local/national	
Hospital Pediátrico, Centro Hospitalar e Universitário de Coimbra		215	60,000	Academic	Inner city	Paediatrician	CTAS	
Emergency Children’s Hospital, Cluj-Napoca	Romania (RO)	168	9400	Teaching	Rural	Paediatrician or PEM	ESI	Yes^b^
Tirgu Mures Emergency Clinical County Hospital, Tirgu Mures		114	16,000	Academic	Inner city	Paediatrician	ESI	
Cruces University Hospital Bilbao, Basque country	Spain (ES)	230	53,000	Academic	Inner city	PEM specialist	CTAS	No
Hospital de Mendaro, Mendaro (Guipúzcua)		60	7160	Non-teaching	Rural	U	U	
Hospital Universitario Rio Hortega, Valladolid		248	24,000	Teaching	Mixed	PEM specialist	PAT	
San Agustín University Hospital, Linares, Jaén		93	U	Teaching	Mixed	Paediatrician	U	
University Hospital, Geneva	Switzerland (CH)	230	25,500	Academic	Inner city	PEM specialist	CTAS	Yes^a^
Children’s Hospital of Zurich, Zurich		198	37,000	Academic	Inner city	Emergency physician	ATS	
Cukurova University Medical Faculty Balcali Hospital, Adana	Turkey (TK)	708	20,000	Academic	Mixed	PEM specialist	None	Yes^b^
St Mary’s Hospital, London	United Kingdom (UK)	145	27,000	Academic	Inner city	PEM specialist	MTS	Yes^b^

^a^Recommended in local triage or ED guideline

^b^Recommended in NICE or NICE-based fever guideline

*PED*, paediatric emergency department; *PEM*, paediatric emergency medicine; *U*, unknown; *MTS*, Manchester triage system; *CTAS*, Canadian triage and acuity scale; *ESI*, emergency severity index; *PAT*, pediatric assessment triangle; *ATS*, Australasian triage scale

### Overall measurement of vital signs and per country

**Fig. 2:**
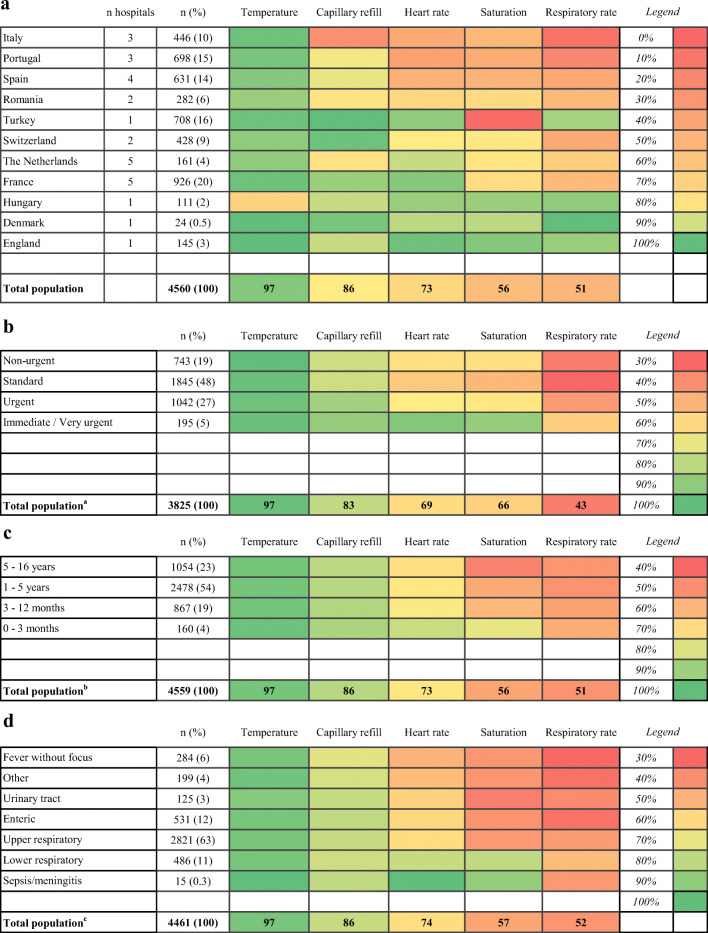
Heat maps indicating the frequency of vital sign measurements in % **a** per country; **b** per triage level; **c** per age group; **d** per diagnosis, Superscript lowercase letters indicate the following: ^a^Turkey (*n* = 708) and 27 other cases excluded for missing triage level; ^b^1 patient missing age; ^c^99 (2%) missing diagnosis. Categories (country, diagnosis and triage level) are ranked from top to bottom according to how often all of the vital signs were measured. Vital signs are in turn organized from left to right based on their frequency of measurement overall. Green indicates highest frequency of measurement per figure; red indicates lowest frequency of measurement

The measurement of vital signs occurred in varying degrees, both when comparing the different vital signs with each other and across participating countries. Overall, temperature was measured most frequently (97%, 4435/4560, 95% confidence interval 97–98%), ranging between countries from 70% (78/111) in Hungary to 100% in Denmark and England (*n* = 24 and *n* = 145 respectively). Capillary refill was next (86%, 85–89%), followed by heart rate (73%, 72–75%), saturation (56%, 55–58%) and respiratory rate (51%, 50–53%), although the latter two had much wider ranges between countries. Figure 2 a contains a heat map visualizing the frequency of vital sign measurements in participating countries. Variability between countries is apparent throughout all of the different vital signs and is most striking for respiratory rates. Temperature was the most consistent, as it was measured in more than 90% of cases in all countries but one.

### Measurement of vital signs per triage level, age group and diagnosis

In the hospital in Turkey, no routine triage was performed. In the remaining hospitals, 99% (3825/3852) of children were triaged upon their arrival to the hospital. Children requiring ‘very urgent’ or ‘immediate’ care had their vital signs measured most frequently (Fig. 2b). Differences across triage levels were greatest for heart rate, saturation and respiratory rate and amounted to about 30% points between the ‘very urgent/immediate’ and ‘standard’ categories (heart rate: 93% vs. 64%, saturation: 90% vs. 59%, respiratory rate: 65% vs. 37%).

Differences in measurement across age groups were smaller (Fig. 2c). Only heart rate and saturation were more frequently measured in infants than in children > 5 years of age (heart rate: 83% vs. 71%; saturation 78% vs. 47%).

We observed an association between the measurement of vital signs and diagnosis (Fig. 2d). Most children (3307/4461, 74%) had respiratory tract infections (RTIs); only 15 children had sepsis or meningitis. Temperature and capillary refill were measured quite consistently across the different infectious foci (97% and 86% of cases respectively), but the remaining vital signs exhibited a considerable variability. Saturation was measured substantially more often in lower RTIs and in patients with sepsis/meningitis than in other cases. Heart rate was recorded in all patients with sepsis/meningitis (15/15) and in 86% (416/486) of those with lower RTIs. For fevers of unknown origin, on the other hand, heart rate measurements were included in the work-up of only 61% (174/284) of cases. Respiratory rates were measured in less than half of patients for four out of seven infectious foci and were done most frequently in patients with lower RTIs, amounting to 64% (310/486) of cases.

### Frequency of abnormal findings

The incidence of abnormal vital signs when measured was generally low. Of all patients with a measured temperature, 2403 (54.2%) had a fever at the time of evaluation in the ED (Table 1). Out of these children, 889 (37%) had a temperature of 39 °C or more. Other than that, heart rate was most often abnormal, in 34.1% of cases. Twenty-nine percent of children were found to be tachypnoeic, hypoxia was found in 3.3% of cases and prolonged capillary refill in 1.5%.

We observed no correlation between the frequency of measurement of a vital sign per country and the proportion of abnormal values (out of all values measured in that country). So, less frequent measurement of a vital sign was not related to a higher proportion of abnormal values detected.

### Adherence to guideline recommendation

From all hospitals using the NICE recommendations, 1450/3014 (48%) of children under five underwent a complete measurement of these vital signs (95% CI 46 to 50%). A complete measurement was most frequent in children with lower RTIs and sepsis, although at a moderate compliance of 55% and 46% respectively (193/350 for lower RTIs and 5/11 for sepsis; Table 3). Multivariable analysis showed that children with RTIs had complete measurements significantly more often than children with fever without focus (odds ratio for upper RTI 1.75 (1.10–2.77), for lower RTI 3.75 (2.21–6.37); Table 3). Also, younger children were more likely to have all recommended vital signs measured than children over 1 year of age. Last, children with high triage urgency had full measurements slightly more often than non-urgent children (immediate/very urgent OR 1.62 (0.95–2.76), urgent level OR 1.36 (0.96–1.95)). Crowding of the ED had no significant effect on the frequency of complete measurement of vital signs. After adjusting for diagnosis, age and triage urgency, a substantial variability between hospitals remained (data not shown).

**Table 3:** Determinants of full measurement of NICE-recommended vital signs in children under five

Full chart measured	n/*N* (%)^a^	OR (95% CI)^b^
Diagnosis		
- Fever without focus	72/170 (42%)	Reference
- Other	53/134 (40%)	0.94 (0.50–1.77)
- Urinary tract infection	37/83 (45%)	1.19 (0.56–2.54)
- Enteric	142/352 (40%)	1.26 (0.75–2.12)
- Upper RTI	922/1856 (50%)	1.75 (1.10–2.77)
- Lower RTI	193/350 (55%)	3.75 (2.21–6.37)
- Sepsis–meningitis	5/11 (46%)	1.93 (0.49–7.65)
Triage level		
- Non-urgent	180/526 (34%)	Reference
- Standard	368/1117 (33%)	0.75 (0.54–1.05)
- Urgent	358/715 (50%)	1.36 (0.96–1.95)
- Immediate or very urgent	98/163 (60%)	1.62 (0.95–2.76)
Crowding of PED		
- Usual number of daily visits	519/1267 (41%)	Reference
- Less visits than usual	168/463 (36%)	0.83 (0.62–1.10)
- More visits than usual	296/775 (38%)	0.98 (0.77–1.24)
Age groups		
- 0 to 3 months	81/139 (58%)	1.76 (1.06–2.92)
- 3 to 12 months	392/728 (54%)	1.38 (1.09–1.75)
- 1 to 5 years	976/2146 (46%)	Reference

^a^Based on population under five from hospitals using NICE recommendations, *n* = 3014

^b^Multivariable analysis, clustered by hospital, based on complete cases, *n* = 2433

*RTI*, respiratory tract infection; *PED*, paediatric emergency department

## Discussion

### Main findings

In this study of febrile children at 28 European EDs, we observed that of all vital signs, temperature is most frequently measured and respiratory rate least frequently, but with a high degree of variability between countries. Most centres have adopted the recommendation of the NICE guideline ‘*Fever in under 5s: assessment and initial management*’ to always measure temperature, heart rate, respiratory rate and capillary refill, but compliance to this recommendation was moderate. Febrile children that are under 1 year of age, with high triage urgency and those with RTIs were more likely to have a full set of vital signs measured.

### Interpretation and comparison to existing literature

Fever was an inclusion criteria for our study, which explains the high frequency of completed temperature in our database and the high proportion of abnormal temperatures. The relatively high proportion with abnormal heart rate can be explained by the physiological relationship between temperature and heart rate [22, 23]. Respiratory rate was least frequently measured and with large variation across subgroups. Other studies have suggested reasons for such variability, like crying or distress of a child, or limitations in the counting technique [11, 24, 25]. We had no information on the child’s well-being or the devices used for measurement of respiratory rate, but these factors may have contributed to the observed low frequency of measurement of this vital sign. Although ED crowding has been associated with decreased quality of care [26], we found no association between ED crowding and adherence to the vital signs measurement recommendation in our study.

We observed an overall adherence of 48% to the NICE recommendation to measure four vital signs in all children under five, in our study in 2014–2016. This is lower than reported by a previous audit study in primary care in the UK (62%) after educational sessions and introduction of a template to record vital signs in the electronic health record [15]. An audit among paediatric EDs in the UK found that temperature was similarly measured as in our study (94%), but reports lower numbers for capillary refill time (53%) and higher rates for heart rate (94%) and respiratory rate (89%) measurements [16]. It may be striking that full measurement of vital signs children under five was most frequently done in children suspected of RTIs, rather than in those with suspected urinary tract infections and fever without focus. Even though the discharge diagnosis is often unknown at the moment of vital sign measurement, it is likely to assume that children with these last two diagnoses might present without specific symptoms. These children may have more diagnostic uncertainty and be at higher risk of complicated disease. Less than half of the children with suspected sepsis—although represented by a small number in our study—received the full set of vital sign measurements needed for compliance with the NICE guidelines.

Patient characteristics can only partly explain the observed practice variations. Professional adherence to guideline recommendations can also be influenced by local policy or professional experience. Even though most participating centres mentioned that their guidelines were based on the NICE guideline, in the process of translation from the UK to another setting, the evidence probably is weighed according to the local setting and practice. This may induce further practice variation across centres [18].

### Strengths and limitations

This study had the advantage of a sizeable, prospectively generated database containing large amounts of high quality patient information from 28 hospitals of various sizes and hospital types, from 11 different countries in Europe. Compared to the available literature in European paediatric emergency medicine, this number of included hospitals and countries is large, supporting the generalizability of our findings. However, some countries and hospitals included more patients than others, which might have influenced results. Furthermore, countries were represented by different numbers of hospitals (some countries only by one hospital), which adds uncertainty to whether measurements are a reflection of national or local policies.

The study was performed in hospitals of the REPEM research collaboration, ensuring high-quality data [27]. Their interest in research indicates that they are likely to uphold a high standard of care. The staff of participating hospitals were only aware of the general study design as a registry of febrile children, so a special focus on vital sign measurement during the study period is unlikely. Lastly, because this research treated missing variables as decisions not to perform certain measurements, some room remains for human error in data collection. However, all items in the data collection form were mandatory, with the option to fill in ‘missing’. During the preparation of this manuscript, the local investigators confirmed that ‘missing’ values were indeed ‘not recorded’.

### Clinical and research implications

Our numbers on compliance to the NICE recommendation obtained from 28 European EDs calls for better recording of vital signs in children. Not measuring vital signs may pose children at risk of underestimating the severity of their illness or delaying necessary treatments [14]. Even though almost all included centres had adopted the NICE recommendation to measure vital signs in all febrile children, compliance in less than half of cases is striking. Even in children with sepsis, fever without source or urinary tract infections in less than 50% of cases the full set was measured. Therefore, special attention should be given to children presenting with fever without specific symptoms, since vital sign measurements may contribute most to the identification of severe infections in this patient group. Although measurement is influenced by age and triage, it might be questioned whether triage appropriately selects children with severe disease [28].

Future research should focus on identifying reasons for non-compliance, including cultural and healthcare factors at the individual, organizational and national level [18]. Qualitative research could provide more in-depth information on the reasons for the observed discrepancies in vital sign measurements across Europe. At the same time, more evidence is needed on the diagnostic value of vital signs in different settings and patient groups and their impact on health outcomes. Such research could provide evidence for targeted measuring of vital signs in children that benefit most from complete measurements.

## Conclusion

Measuring vital signs in children with fever in the emergency department occurs with a high degree of practice variation between different European hospitals and is done more often in younger children, those with a higher triage urgency or who have respiratory tract infections. The overall adherence to the NICE recommendation to measure four vital signs in all febrile children under five is moderate. Our practice variation study is essential as a benchmark for current clinical practice. It can guide future research into the drivers and consequences of the observed under-recording of vital signs. Moreover, it can be used to tailor implementation strategies of the NICE recommendation to different European settings.

## Electronic supplementary material

ESM 1 (PDF 114 kb)

ESM 2 (PDF 101 kb)

## Abbreviations

APLS: Advanced pediatric life support
CRF: Case report form
ED: Emergency department
NICE: National Institute for Health and Care Excellence
PEWS: Paediatric early warning scores
REPEM: Research in European Pediatric Emergency Medicine
RTI: Respiratory tract infections
SBI: Serious bacterial infections
UK: United Kingdom
