# Supplementary material for: Measuring vital signs in children with fever at the emergency department: an observational study on adherence to the NICE recommendations in Europe
Source: Eur J Pediatr. 2020 Feb 8;179(7):1097–106. doi: 10.1007/s00431-020-03601-y (PMC7314716; doi:10.1007/s00431-020-03601-y)
Supplement: Supplementary file 1 — (PDF 114 kb) [file 431_2020_3601_MOESM1_ESM.pdf]

Addendum 1. To 'Antibiotic prescription in febrile children: impact of clinical profile and cultural background', Oostenbrink et al

Patient data collection (to be completed for each patient at registration day 7.30 a.m. – 7.30 a.m. following day)

General questions/Hospital data

Code of hospital, name and country (in final webform each participating hospital will be included in a list where to choose from)

Gender child (male/female/not known)

Referral (GP/self/ambulance/other/not known)

Weight of child (kg/ xxxx if unknown)

Comorbidity (none/cardiac/pulmonary/immunodeficiency/neurologic/other/unknown)

Date of visit (ddmmyyyy)

Date of birth (ddmmyyyy)

Hour of visit (00.00h (24 hours time))

Total number of pediatric emergency visit (7.30 a.m – 7.30 a.m following day) at registration day (number/ 999 if unknown)

Specific disease questions

Triage code (immediate, very urgent, urgent, standard, non-urgent/unknown)

Duration of fever (days(rounded to half)/ 999 if unknown)

Ill appearance (yes/no/unknown)

Heart rate (per minute/999 if unknown)

Respiratory rate (per minute/999 if unknown)

Cutaneous saturation (%/999 if unknown)

Body temperature (°C/ 99 if unknown)

Peripheral capillary refill (normal = <2sec/prolonged = 2-4 se/very prolonged = >4 sec/unknown)

Consciousness (awake, responsive to speech, responsive to pain, non responsive /unknown)

Increased work of breathing (defined as presence of chest wall retractions/nasal flaring or grunting) (yes/no/unknown)

Petechiae (yes/no/unknown)

Meningeal signs (defined as presence of Kernig, Brudzinsky, tripod phenomenon, bulging fontanelle for <1 year) (yes/no/unknown)

Seizure (generalized jerks with consciousness loss) (yes/no/unknown)

Diagnostic

CRP performed (yes/no/unknown)

value (mg/L/ NA/9999 if unknown)

Leukocyte count performed (yes/no/unknown)

Value (*10^{9}/L/NA/999 if unknown)

PCT performed (yes/no/unknown)

Value: (ng/L/ NA/9999 if unknown)

Chest X-ray performed (yes/no/unknown)

If yes: what is result (normal/local infiltrate/diffuse abnormalities/other/NA/unnown)

Urinalysis performed (yes/no/unknown)

If yes: what is result (leuocyturia yes, nitrite positive yes/nitrite and leukcyturia present/none present/NA/unknown)

Bloodculture performed (yes/no/not known)

If yes: result:…………………………………….

Lumbar puncture performed (yes/no/unknown)

If yes: result:…………………………………………………..

Treatment

Antibiotics prescribed (yes/no/unknown)

If yes, type/name:……………………………………

If yes, dose and frequency (mg/times/day)

Prescription way (oral/ intravenous/intramuscular/unknown)

Main motivation for antibiotic prescription:

Clinical symptoms (yes/no/unknown)

Results of laboratory tests (yes/no/unknown)

Parental request (yes/no/unknown)

Based on guideline advice (yes/no/unknown)

Other reasons:……..

Evolution

Destination (discharged home/ discharged after observation unit <24h/ admission to ward/ admission to ICU/ dead in ED/unknown)

Working diagnosis (Definite bacterial /Probable bacterial / Definite viral/probable viral/uncertain)

Focus (upper respiratory/lower respiratory/ urinary tract/ enteric/ cutaneous/ sepsis-meningitis/ other/fever without focus/unknown)

If other, specify:……………………………………
