# Supplementary material for: Measuring vital signs in children with fever at the emergency department: an observational study on adherence to the NICE recommendations in Europe
Source: Eur J Pediatr. 2020 Feb 8;179(7):1097–106. doi: 10.1007/s00431-020-03601-y (PMC7314716; doi:10.1007/s00431-020-03601-y)
Supplement: Supplementary file 2 — (PDF 101 kb) [file 431_2020_3601_MOESM2_ESM.pdf]

Addendum 2. To 'Antibiotic prescription in febrile children: impact of clinical profile and cultural background', Oostenbrink et al

Hospital data collection (to be completed once for the study)

General questions/Hospital data

Data of registration (ddmmyyyy)

Code of hospital, name and country (in final webform each participating hospital will be included in a list where to choose from)

Adherence area (number/completed by 9.999.999 if unknown)

Setting (inner city/ rural/mixed/not known)

Type (academic/teaching/ non-teaching/not known)

Number of pediatric emergency care admittance annually (number/completed by 999.999 if unknown)

Type of triage system used (none/MTS/CTCS/ESI/other/not known)

If other: please specify name:……………………………

Availability of specific guidelines:

a) Guideline for children with fever (NICE/local/other/none/not known)
    a. If local or other, please specify:……………………………
b) Guideline for children with urinary tract infections (NICE/local/other/none/not known)
    a. If local or other, please specify:……………………………..
c) Guideline for children with respiratory infections (British thoracic society/NICE/local/other/none/not known)
    a. If local or other, please specify:…………………………………
d) Guideline for children suspected for meningitis/sepsis (NICE/local/other/none/not known)
    a. If local or other, please specify:………………………………..
e) Other guidelines (yes/no/not known
    a. If other, please specify:……………………………………………

Description of national immunization schedule

a) National immunization coverage: …%
b) DKTP availability yes/no; administration schedule….; name vaccine:…
c) HIB availability yes/no; administration schedule….; name vaccine:…
d) S. Pneumoniae availability yes/no; administration schedule….; name vaccine:…
e) N. Meningococcus C availability yes/no; administration schedule….; name vaccine:…
f) Hepatitis B availability yes/no; administration schedule….; name vaccine:…
